# Tapered insect (*Acheta domesticus*) antennae have rapid damped return with minimal oscillation after perturbation

**DOI:** 10.1242/jeb.249243

**Published:** 2025-05-06

**Authors:** Marlo G. McCarter, Derek Kellogg, Stanley Sowy, Catherine Loudon

**Affiliations:** ^1^ Department of Ecology and Evolutionary Biology, University of California, Irvine, Irvine, CA 92697-2525, USA; ^2^ Department of Ecology and Evolutionary Biology, University of Kansas, Lawrence, KS 66045, USA

**Keywords:** Sensory, Biomechanics, Damping, Orthoptera, Vibration, Mechanosensory

## Abstract

As tactile sensors, antennae must be flexible and responsive while maintaining shape and control of the structure. We evaluated the geometric and mechanical properties of cricket antennae, which we treat as bending cantilever beams. Flexural rigidity (*EI*) is the mechanical property that most significantly controls bending behavior. We determined that the flexural rigidity decreases steeply (proximal to distal) by evaluating the quasistatic bent shapes in response to obstacle contact at different points along the antennae. This steep decrease in flexural rigidity causes the antennae to bend readily only near the obstacle contact, in contrast to the curvature of a beam with uniform properties and cross-section (which bends closer to the base). This flexural rigidity gradient in the antennae is consistent with the morphology: a decreasing second moment of area calculated from the measured taper and the diminishing wall (cuticle) thickness. Cricket antennae recovered from a single localized perturbation quickly and with minimal to no oscillation, suggesting behavior close to critical damping (fastest return without oscillations). Bending primarily occurred in the portion of the flagellum near the obstacle contact, reducing the length of the flagellum that participated in the oscillating behavior (natural frequency ∼11 Hz). Forced sinusoidal vibrations generated a resonance frequency of ∼30 Hz with imperceptible movement in the proximal part of the flagellum while the distal part vibrated. The results suggest that tapering of an elongated mechanosensor may facilitate a rapid return to its original shape without oscillation, which is an advantageous attribute that may also inform biomimetic applications.

## Teaser

**Summary:** Cricket antennae are long tapering sensory structures that return rapidly to their resting shape with minimal to no vibration after contact, facilitating a quicker response to the next mechanical stimulus.

## INTRODUCTION

**Fig. 1.**
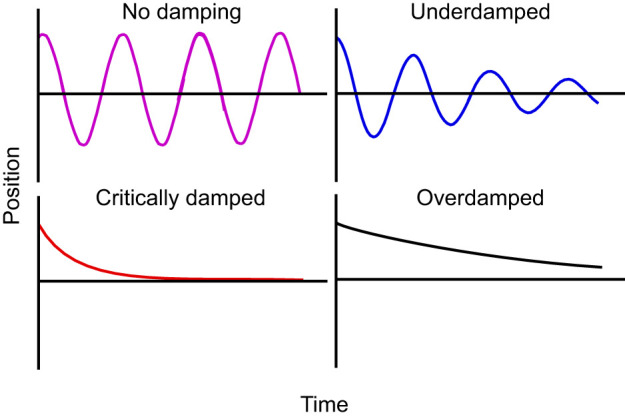
**Illustration of theoretical oscillation with categories of damping after deflection.** Position of a beam's unfixed end (*y*-axis) as it releases stored elastic energy from the deflection by recoiling to its original shape, over time (*x*-axis). As the damping increases, oscillations by the structure decrease.

Many insects have long, thin and tapering antennae that they use for tactile evaluation of their environment (Harley et al., 2009). Antennae are composed of three main segments: the scape, which is attached to the head, followed by the pedicel and the longer sub-segmented flagellum (Chapman et al., 2013). Antennae readily deflect and bend, and information about the displacement of an antenna is received by two sensory organs located at the scape and the pedicel (Comer et al., 2003; Loudon, 2009; Okada and Toh, 2000; Sane et al., 2007). Bending occurs primarily at joints between segments or subsegments (Loudon et al., 2014; Schneider, 1964), and the antenna as a whole can be actively moved by muscles that run from the head capsule into the scape, and from the scape to the pedicel (Chapman et al., 2013; Dürr et al., 2001). Greater length of an antenna allows for greater reach but could also decrease tip positioning control and increase vibration (Dirks and Dürr, 2011; Yang et al., 2019). Damping is the dissipation of the stored elastic energy that drives the movement following a deflection, thereby reducing the oscillation of the structure. Damping can result from external forces acting on the moving antenna (e.g. drag) or internal effects (e.g. energy dissipation of viscoelastic materials or fluid movements within the antenna). Damping can be categorized into three levels: underdamped, critically damped and overdamped (Fig. 1) (Beer et al., 2016). Underdamping is when the stored energy within an antenna dissipates slowly during an elastic recoil, resulting in many oscillations before the structure loses enough energy and movement stops. In critical damping, energy dissipates more rapidly, such that the structure returns as quickly as possible to its original position, without oscillating. Overdamping results when energy dissipates even more rapidly than in the case of critical damping, resulting in a slower or even incomplete return of the structure to its original shape without more energy input. In practice, experimental resolution will limit identification of a return to an equilibrium state (Lelas et al., 2023). A mechanosensory structure would be expected to operate close to critical damping if residual oscillations interfered with the detection of new stimuli (Droogendijk et al., 2014; Miles et al., 1995; Robert et al., 1996). As there is no simple predictive relationship for critical damping for the relevant geometry (tapered cantilever), we used an empirical approach to evaluate whether cricket antennae reconfiguring after perturbation were operating close to critical damping. In addition, we evaluated antennal static bent shapes to estimate the longitudinal gradient in flexural rigidity (*EI*) that would be expected for a tapered structure and could facilitate the damping behavior.

## MATERIALS AND METHODS

### Experimental animals

Crickets of the species *Acheta domesticus* (Linnaeus 1758) were from Fluker's Cricket Farm (Port Allen, LA, USA). Only adults were used, of both sexes. All measurements were made on live, restrained crickets.

### Antennal geometry

**Fig. 2.**
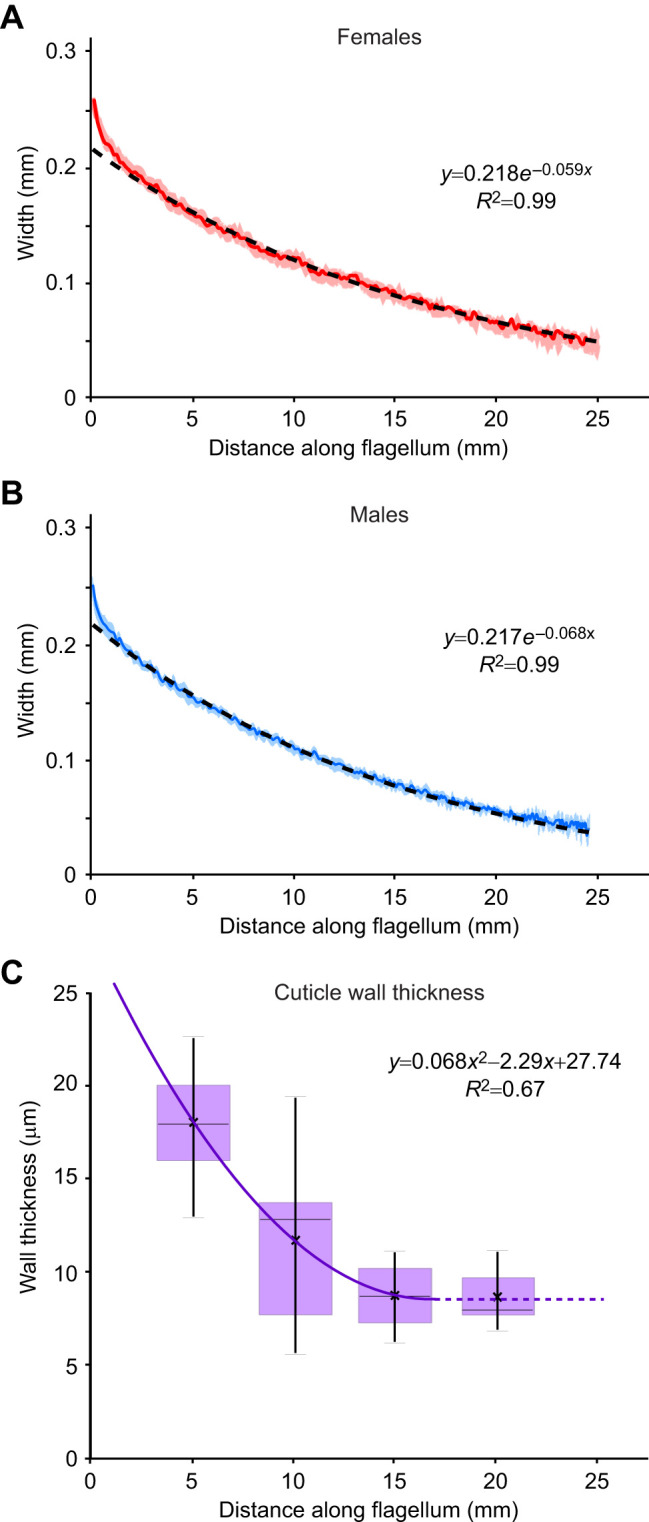
**Antennal geometry in adult crickets showing taper.** (A,B) The average width for each flagellomere (with 95% CI) is plotted against distance along the flagellum (summed average flagellomere lengths to that point) for each flagellomere (*N=*15 pairs of antennae for each sex, ∼300 flagellomeres per antenna) for females (A) and males (B). The dashed lines show the exponential equations fit to the means. (C) Wall thickness is plotted against distance along the flagellum (*N*=5 males, 5 females), means indicated by ‘x’, medians by horizontal lines, the interquartile range is shown by the box (purple) and whiskers display the entire range of the data. A regression line is shown in purple; the dashed portion of the line indicates where the wall thickness no longer decreases and is treated as a constant. Lengths and widths for all flagellomeres from Loudon et al. (2014) were used to calculate the taper along the length of the flagella.

Lengths and widths for all flagellomeres (∼300 per antenna) of the antennae from 15 male and 15 female adult crickets from Loudon et al. (2014) were used to calculate the taper along the length of the flagella (Fig. 2). Flagellomere measurements from the left and right antennae from any individual were averaged together before averaging between individuals. An exponential curve was fit to the data; this provided a better fit than polynomials (second or third order), as determined by examining the pattern of the residuals. Antennal morphology was not normalized (see Fig. S1 for justification). To evaluate the possibility of differences in antennal morphology between these earlier measurements and the current cricket population, additional measurements of antennal widths from five male and five female adult crickets were taken from the current cricket population at distances of 5, 10, 15 and 20 mm along the flagella (measured from the pedicel/flagellum border). There was no significant difference between the current crickets and the predicted fit at distances of 5 or 10 mm, although the current cricket antennae were slightly wider at 15 mm (11%, *N*=10) and 20 mm (20%, *N*=10) (one-sample *t*-test comparing against the predicted value from the exponential fit for that distance, males and females combined: 5 mm, *P=*0.14, *N*=10; 10 mm, *P*=0.30, *N*=10; 15 mm, *P*<0.001, *N*=10; 20 mm, *P*<0.001, *N*=10). This minor difference would only produce a small change for more distal deflections.

The wall thickness was roughly estimated at different locations along the flagella; one antenna was removed from each of five male and five female adult crickets and cut in cross-section at distances of 5, 10, 15 and 20 mm from the pedicel–flagellum boundary with a sharp scalpel. The pieces were mounted on carbon tape on pieces of foil attached to SEM stubs, sputter-coated (7 nm Pt/Pd, Leica ACE200, Deerfield, IL, USA), and viewed end-on in an electron microscope (FEI Quanta 3D FEG FIB/SEM, Waltham, MA, USA). The SEM images were opened in ImageJ/Fiji (Schindelin et al., 2012), and the outer edge and inner edge were manually traced using the segmented line tool. We used the traced edge lengths to estimate the outer circumference (C*_i_*_,out_) and inner circumference (C*_i_*_,in_). The nominal wall thickness (*w*) was estimated using the simple case of a circular cylinder, because the flagella are approximately circular in cross-section:
(1)

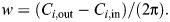


### Video recordings

**Fig. 3.**
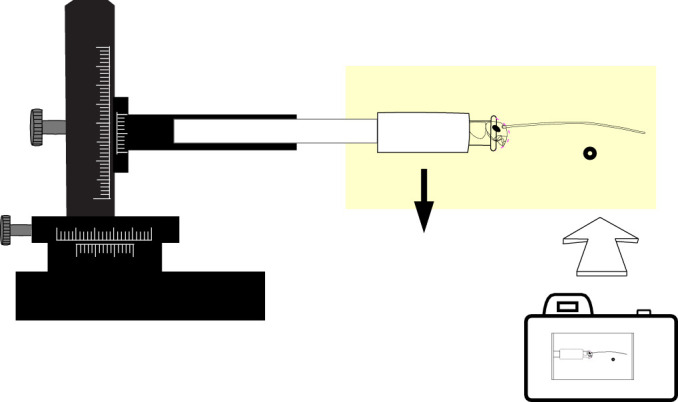
**Experimental setup for deflection of cricket antennae.** Crickets were filmed from a side view with the obstacle (glass microcapillary tube) perpendicular to the antenna. Camera field of view illustrated by the yellow shading. The black arrow shows the movement of the cricket to generate an upward deflection.

CO_2_ was used to temporarily immobilize live crickets (10 male and 10 female adults), which were then carefully inserted into microcentrifuge vials (0.6 ml, polypropylene, Fisherbrand, Thermo Fisher Scientific) up to their head capsule, restricting their movement. Vial ends were cut to allow airflow into the tube for continued cricket respiration. One antenna was removed, and the head capsule was enclosed in epoxy (5-min quick-setting epoxy, J-B Weld, Marietta, GA, USA), including the scape, pedicel and the first few flagellomeres of the remaining antenna, to restrict the live cricket from moving the antennae with its muscles. A low stream of CO_2_ supplied continually to the open end of the vial prevented the cricket from moving its antenna from a straight forward position during the application and drying of the epoxy. The epoxy was extended to the edge of the microcentrifuge vial, which ensured the head was firmly anchored in the vial. Crickets were verified as alive by observing the movement of their legs or ovipositor through the transparent vial before the vial was inserted into a hollow rod. The rod was positioned and smoothly moved up and down with a manual 3D micromanipulator, such that the antennae contacted the obstacle, a smooth glass micropipette (50 µl borosilicate glass micropipette, 1.4 mm outer diameter, Fisherbrand, Thermo Fisher Scientific), at a right angle and slipped past it (Fig. 3; Movie 1). Once the antenna began to slip with respect to the obstacle, we stopped moving the micromanipulator to allow the antennae to passively slip off. Analyzing the return following a deflection can be used to determine the damping characteristics of a cantilever beam (Dudek and Full, 2006; Hartmann et al., 2003; Tongue, 2002).

The antennal deflections and returns were recorded at 200 frames s^−1^ (Sentech camera STC-MCCM200U3V, Goya MP Resolution lens). Preliminary recordings made at 488 frames s^−1^ showed that 200 frames s^−1^ would be a sufficient recording frequency to capture the oscillations of the tip. The antennae encountered the obstacle at four distances from the base of the flagellum (5, 10, 15 and 20 mm), positioned using the micromanipulator and alternated starting at the proximal or distal end between individuals. Antennae from two crickets (one female and one male) were shorter than 20 mm and therefore only three distances were recorded for those two crickets. We alternated between down and up movements, recording three deflections in each direction of movement, for each distance (a total of 24 videos per cricket, 18 videos for the two crickets with shorter antennae).

### Digitizing

**Fig. 4.**
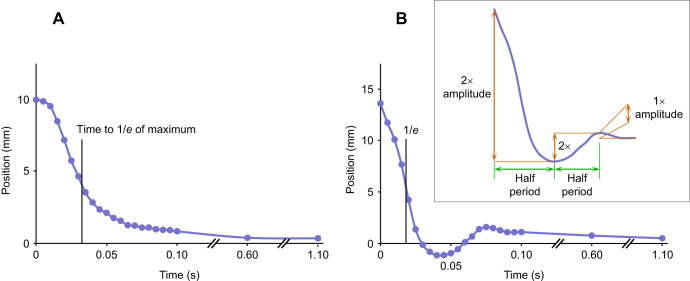
**Change in vertical position of the antennal tip after slipping past an obstacle (time=0) and returning to its original position.** A position of zero is the tip position prior to the deflection, with the initial deflection displayed as a positive *y* to facilitate comparison between deflections in different directions. (A) An example time course of the tip returning to the original position without oscillating after the antenna was deflected downwards by the obstacle located 10 mm from the proximal end of the flagellum. (B) An example time course of the tip returning to the original position in which the tip showed two reversals in direction (1.5 cycles of oscillation) after being deflected upwards by the obstacle located 5 mm from the proximal end of the flagellum. The inset shows how the amplitudes and periods were measured for the oscillations when both are changing over time. Markers indicate digitized coordinates (no filtering). The time to return to 1/*e* of the maximum vertical position is indicated on both graphs.

The *x* and *y* coordinates of the base and the tip of the antenna (in the frame of reference of the camera) were manually digitized from each video using MaxTRAQ version 2.9.1.8 (Innovision Systems Inc., Columbiaville, MI, USA). The digitized frames included before deflection, for each frame during the deflection, for the return until the antennae no longer oscillated (usually about 0.1 s after the antenna had slipped past the obstacle), and an additional 0.5 and 1 s later (Fig. 4). Each frame had a resolution of 1024×768 pixels, which corresponded to a spatial resolution of 11.7 pixels mm^−1^ (real world) for the field of view. The digitized coordinate locations of the obstacle were used for calculations but were categorized into the four real-world measured distances (5, 10, 15 and 20 mm) for statistical analyses. To estimate the variability in antennal tip resting position for a single cricket, we additionally digitized the antennal tip both before and after a deflection for all six videos for a single distance (three replicates in each direction) for each of six crickets (three males and three females) (s.d.=0.6 mm, *N*=36).

Although MaxTRAQ allows for interpolation to subpixel sampling, a single pixel was used as a conservative estimate of uncertainty in the measurement of the *x* and *y* coordinates (e.g. Taylor, 1997). We corroborated the digitizing uncertainty by repeatedly digitizing the base and tip of one antennal image, independently 10 times. The sample standard deviation of *x* coordinates, *y* coordinates and the 2D distance between the two points were all <1 pixel, which suggests that an uncertainty of 1 pixel (85.5 μm) was appropriate.

### Calculations of amplitude, time constants and natural frequency

Amplitude and time constants were calculated using custom scripts and MATLAB (MathWorks, Inc., Natick, MA, USA). To evaluate the deflections of the antenna, the *x* and *y* coordinates for the antennal tip were transformed for each frame relative to the simultaneous coordinates of the (moving) antennal base, such that the starting rest position of the tip (relative to the antennal base) had a *y* coordinate of zero. Oscillation amplitudes (identified by reversal of the tip movement) were estimated from the distance between a peak and an adjacent trough (Fig. 4B). When there was no subsequent peak or trough, the amplitude was estimated relative to the location of the stationary antennal tip following the deflection (e.g. time of 1.1 s; Fig. 4B). A peak or trough was identified from the time course of the raw data as two successive increases in the digitized height of the tip (*y*) in two successive frames followed by two successive decreases (peak), or two successive decreases followed by two successive increases (trough) in our custom MATLAB script. Movements of the antennal tip starting after two standard deviations of the average time to the first tip reversal in direction typically had a small amplitude, were interpreted as noise, and therefore were not counted as relevant oscillations. The minimum detectable period for oscillation was 0.02 s (for our frame rate of 200 frames s^−1^). To calculate natural frequency (*f*), we used the reciprocal of the period (*T*):
(2)

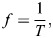
where *f* is frequency in Hz, and *T* has units of seconds. The period was determined from the time between digitized peaks and troughs (see Fig. 4 for more details) with 0.05 s resolution (200 frames s^−1^). The average period of cricket deflections was used.

To characterize the time to elastically return to the resting position, we measured the time constant τ*_e_* (the time to reach 1/*e* of the difference between the maximal deflected position and the location of the stationary tip following the deflection; Fig. 4A), which is an approach that approximates the movement as a simple exponential decay (Dirks and Dürr, 2011). For those antennal tips that showed oscillation (e.g. Fig. 4B but not Fig. 4A), the natural frequency of those antennae was calculated from the periods of those oscillations.

### Estimate of out-of-plane bending

To evaluate whether any of the bending of the antennae was out of the plane of view (into or out of the image), the apparent length of the antennae was traced from still images before bending and during bending, using the ImageJ segmented line tool. Twenty pairs (half male, half female) of the length measurements were analyzed for the two most proximal deflection contact distances (5 and 10 mm from the base of the antenna), as more of the antenna participates in these deflections, increasing the chance of out-of-plane bending occurring.

### Estimation of zeta

We determined the logarithmic decrement, delta (δ), describing the decrease in amplitude (*P*) after *n* cycles:
(3)

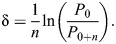


Eqn 3 is particularly reliable when there is a constant exponential decay in the amplitude. If exponential decay in amplitude is not constant, using fewer cycles (e.g. *n*=1) is more accurate. The logarithmic decrement may be used to calculate the damping ratio, zeta (ζ), the ratio of the damping present to critical damping (Tongue, 2002):
(4)

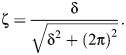


For more details on the use of this approach, see Hartmann et al. (2003).

### Resonance frequency of cricket antennae

To determine the resonant frequency of the antennae, we applied sinusoidal vibrations to live crickets constrained in tubes with epoxy-fixed antennal joints (identical preparation to those crickets used for single antennal perturbations). The sinusoidal vibrations were applied at different frequencies in increments of 10 Hz from 10 to 100 Hz using a shaker table (model ED-1 Electrodynamic Shaker, Crowson Technology, Santa Barbara, CA, USA) driven by a sweep function generator (model 4003A, BK Precision, Yorba Linda, CA, USA). The shaker table moved up and down, with the frequency confirmed by an accelerometer mounted on the top of the shaker table (model CV2260-050, Crowson Technology) with output sent to an oscilloscope (20 MHz Dual Trace Oscilloscope model 72-6805, Tenma Test Equipment, Springboro, OH, USA). The amplitude of the vibration of the table (half of the up–down translation during the sinusoidal movement) was imperceptible at the resolution of the videos; the amplitude of the sinusoidally varying acceleration was kept constant at 0.62 m s^−2^, which is consistent with a very small translation (a corresponding calculated theoretical amplitude of 42 μm at 20 Hz). High-speed videos (500 frames s^−1^) of the antennae during the oscillations were recorded to determine the frequency response of the amplitude of the antennae for each test frequency; the driving vibration frequency was kept constant for several seconds before collecting data at each test frequency. Total length, length of vibrating portion and the displacement of the antennal tip were measured from digitized still images taken from recorded videos using the straight-line tool (Canvas GFX, Inc.) for a single cycle.

### Estimate of flexural rigidity (*EI*) gradient from shape during bending

Flexural rigidity is the primary property that affects the bending of a beam and is the product *E×I*. *E* (Young's modulus) is the stiffness of the material, and *I* is the second moment of area, or how the area of mass is distributed in the cross-section perpendicular to the bending axis (note that ‘flexural rigidity’, used in the engineering literature, Beer et al., 2020, p. 604; Gere and Timoshenko, 1997, p. 313, is synonymous with ‘flexural stiffness’, which is often used in the biomechanical literature; Vogel, 2013; Wainwright et al., 1976). There is not a closed-form solution for the differential equations for the deflection of a tapered beam (Silverman and Farrah, 2018), so we made estimates by analyzing the shape during bending. To estimate the nominal flexural rigidity (*EI*) gradient of the antennae, we compared the shape of real antennae bent under loading with finite element model (FEM) simulated deflections assuming different flexural rigidity gradients. Still images (1024×768 pixels) were taken from high-speed videos of the antennae both prior to any deflection and during contact with the obstacle for a subset of 10 crickets (five males, five females), chosen using a random number generator. A total of 80 pairs of images (each pair is undeflected/deflected) were used for this quasi-static analysis (10 crickets×4 distances×2 deflection directions). Bending behavior can be more accurately estimated for small deflections, so we used video frames in which the antennae were moderately deflected, taking the images from roughly the temporal halfway point between first contact with the obstacle and the antennae at full deflection. For these images, the average ratio of the curved length to the straight distance between the base of the flagellum and the contact point was 1.24±0.22 (mean±s.d., *N*=80), demonstrating a modest curvature. Elastica 2D assumes quasi-static conditions, and therefore we used frames when the cricket was not moving at all (the undeflected shapes) or very slowly (the deflected shapes); the downward or upward velocity of the cricket corresponding to the time of the deflected shape images used was 2.1±1.04 mm s^−1^ (mean±s.d., *N*=80, measured over 0.1 s interval centered on the frame used). Elastica 2D is an FEM of a beam that consists of ‘elements’ (which act as rigid links) connected by ‘nodes’ (which behave as torsional springs) (Quist and Hartmann, 2012). The shapes of the antennae were traced using a curvilinear line tool, a grid was overlayed on the traced line to divide the trace into individual elements using Canvas X (Canvas GFX, Inc.), and the coordinate points for the nodes were exported.

Elastica 2D is quasi-static and uses an iterative numerical approach to simulate bending the antennae until it meets the obstacle coordinates. The undeflected shape of each antenna served as the input starting shape. To determine the change in shape during deflection, the model uses the relationship between change in the local curvature between points (d*k*), local bending moment of the beam (*M*) and local flexural rigidity (*EI*):
(4)

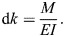


The change in curvature is also related to the change in arc length (d*s*) and the change in angle (dθ) between nodes:
(5)

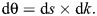


Therefore, the change in angle between two nodes is determined by:
(6)

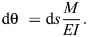


Elastica 2D iteratively determines the change in curvature for each node while finding a stable configuration for the bent (deflected) antenna (see Supplementary Materials and Methods for more details). Flexural rigidity (*EI*) is used to calculate local curvature and bending moment for each node. We simulated deflections of antennal models with four different longitudinal gradients in flexural rigidity: (1) a solid cylinder of uniform radius, (2) a solid cylinder with a linearly decreasing taper, (3) a solid cylinder with an exponentially decreasing taper and (4) a hollow exponentially tapering cylinder with a decreasing wall thickness (all models used parameters from measured antennal geometry; see below for more details). For the simulations, we treated *E* as a constant (our empirical estimate of *E*), and only varied *I* (based on geometry) along the length of the cylinder to generate the longitudinal gradient for *EI*.

For the first hypothetical flexural rigidity gradient (constant *EI* along the length), we used an arbitrary value of 1 for *I* (the output shape is independent of the magnitude of a constant *EI*). For the second hypothetical flexural rigidity gradient (linear taper), we used the morphology determined by combining the measurements for males and females; therefore, the linear taper had a radius of 0.125 mm at the base, and a radius of 0.02 mm at a distance of 25 mm from the base. The second moment of area (*I_i_*) was then calculated at each node using the equation for a cylinder with a circular cross-sectional area, with *r_i_*_,outer_ corresponding to the radius at the same node along the length of the antenna for the linear taper:
(7)

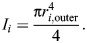


For the third hypothetical flexural rigidity gradient (exponential taper), we used the exponential curve fit to the morphological measurements (Fig. 2). As before, the second moment of area (*I_i_*) was then calculated at each node using Eqn 8.

For the fourth hypothetical flexural rigidity gradient, the antenna was modeled as an exponential tapering hollow cylinder with an inner wall radius (*r_i_*_,inner_) and thinning outer wall radius (*r_i_*_,outer_). The inner radius was determined at each node using the equation for change in wall thickness of the antennae as a function of the position along the length of the antenna. The fitted curve is wall thickness (µm)=(0.0621×(*d*^2^)−2.172×*d*+27.29), where *d* is the distance (mm) along the antennae (Fig. 2). The inner radius was determined by subtracting the wall thickness from the outside radius. Using this formula, the minimum wall thickness (0.008 mm) is reached at a distance of 16 mm from the base and assumed to be constant beyond this point. A single outlier (greater than 1.5×interquartile range) was removed. We calculated local *I_i_* for each point along the cylinder using the equation for a hollow cylinder cross-section at each point on the antennae:
(8)

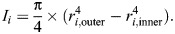


Each of the actual bent (quasi-static) antennal shapes (*N*=80) was compared with the four simulated bent shapes for the same antenna (the four *EI* gradients), using the area between the actual and simulated shapes to estimate the fit (approach also used in Birdwell et al., 2007). The simulated shapes were then ranked (1–4) by how closely they matched the corresponding actual shape, with 1 being the closest fit and 4 being the worst fit. The areas between the actual shape and the simulated shape from the antennal base to the obstacle were determined using the MATLAB function ‘polyshape’ to create a polygon with a measurable area between the two curves. For our mixed model analysis, we normalized the areas for the different *EI* gradients with respect to the area for the hollow exponential *EI* gradient to address the large differences in variation (area other *EI* gradient–area hollow exponential *EI* gradient)/(area hollow exponential *EI* gradient).

### Estimate of material stiffness (*E*)

To include an estimate of *E* in our model, we used a point-load deflection method (Tongue, 2002). For a cantilever beam of length *L*, a point load of known weight (*P*) can be applied at a known distance from the supported end (*a*), and the deflection distance of the tip of the beam (*D*) can be used to determine *EI* of the loaded part of the beam. For a known *I*, *E* can then be solved using the equation (Gere and Timoshenko, 1997):
(9)

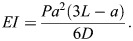


Small weights were made from short lengths of wire (maximum 3.6 mg and minimum 0.716 mg) and fashioned into hooks to hang on the antennae. The weights were placed on the antennae at distances between 5 and 12 mm from the base of the flagellum for seven individual cricket antennae (total of 13 deflections). Weights could not reliably be applied beyond 12 mm without the antennae bending too much to support the weight. The application of the weights was recorded at 200 frames s^−1^ and the deflection distance *D* was measured from still images in Canvas GFX. A rough estimate of *E* was generated for the loaded section of the antennae from Eqn 9 using an average *I* over the loaded section (assuming the exponentially tapered hollow geometry described above). This resulted in an average nominal *E* of 0.2±0.5 GPa (*N*=13). We used this estimate to refine and validate an estimate of *E*, using the FEM script Elastica 2D (described above, but now in ‘force mode’) to iteratively estimate *E* using 0.2 GPa as a starting value, and adjusting the value of *E* up or down incrementally (by 1 MPa for each iteration) until the predicted tip displacement matched the measured tip displacement (within the uncertainty of the incremental changes) given the known point force.

### Statistical analysis

Statistical analyses were performed in SAS 9.4 (SAS institute Inc., Cary, NC, USA). Stepwise addition of variables in linear mixed model analyses (Harrison et al., 2018) used Proc Mixed. Individual cricket identity was included as a random effect, removed as an included effect if not significant, and Type III test results were reported. Frequencies were evaluated using the chi-squared statistic in Proc Freq with cricket as a repeated measure (Stokes et al., 2000). ANOVA analyses were performed on frequency and vibrating length of the antennae. The *post hoc* comparisons between estimates of fit for antennal shapes (based on different assumptions of the longitudinal *EI* gradient) used Proc Mixed with the LSMEANS Tukey option to adjust the *P* for multiple pairwise comparisons.

## RESULTS

### Geometry of antennae

We analyzed the widths of individual flagellomeres along the length of antennae to quantify the tapering (*N*=30 antennae). The width of the antennae decreases exponentially from proximal to distal for both males and females (Fig. 2A,B); there is a slight but significant difference in the exponent of the two sexes (ANOVA, sex: *F*_1,28_=12.69, *P*=0.0013, *N*=30). This small difference (−0.059 versus −0.068; Fig. 2) is expected to have a negligible effect on bending behavior, and therefore we pooled the data in subsequent analyses. The combined male and female equation for exponential taper is *w*=0.2137*e*^(−0.06240*x*)^ (*R*^2^=0.9), where *w* is the antennal width (diameter, mm) and *x* is the distance (mm) along the length of the flagellum from the base (pedicel–flagellum boundary). Antennal wall thickness decreases longitudinally (Fig. 2C), and there was no significant difference in wall thickness in the two sexes (mixed model, distance: *F*_3,31_=24.11, *P<*0.0001, sex: *F*_1,31_=0.19, *P*=0.61; distance×sex: *F*_3,31_=1.75, *P*=0.60; *N*=39); statistical results were comparable regardless of whether distance was treated as a covariate or a fixed effect, and whether a single outlier was included.

### Estimates of antennal material stiffness, *E*

**Fig. 5.**
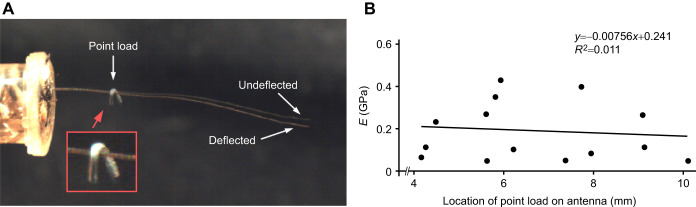
**Stiffness (*E*) estimated from deflections of the tip of the antennae in response to point loads.** (A) Superimposed images of cricket antenna with and without the point load showing the magnitude of the deflection. (B) Estimates of *E* plotted against the location of the point load (measured from the proximal end of the flagellum). The regression slope was not significantly different from 0 (*P*=0.73, *N*=13 measurements from 7 crickets). The units for the regression equation are GPa and mm for *y* and *x*, respectively.

The average nominal *E* for the loaded section of antennae in response to a static point load was 0.2±0.1 GPa (mean±s.d., *N*=13 deflections). There was no trend in measurements of *E* along the length of the antennae (slope of linear regression not significantly different from zero; Fig. 5), similar to estimates of *E* made by Mongeau et al. (2014) for cockroach antennae. Estimates of *E* made in this way are nominal approximations, because they include aspects of both material and structural properties, and complex biological materials can be anisotropic and nonuniform (Birdwell et al., 2007).

### Return to original shape after deflection

To determine whether single or successive deflections plastically deformed antennae, we compared the starting position of the antennal tip with its post-deflection position. We alternated three upward deflections with three downward deflections, with the obstacle at a single distance from the base of the flagellum. The results showed that antennal tips were only 0.2±0.6 mm (mean±s.d.) from their original position in subsequent deflections (*N*=30 for six crickets, five subsequent deflections, obstacle 5 mm from base), which were approximately 30 s apart. Within six successive deflections, there was no trend in the order. Antennae stayed slightly displaced in the direction they were just deflected; an upward deflection resulted in a lingering upward displacement of an average of 0.4±0.6 mm, and a downward deflection resulted in a lingering downward displacement of 0.2±0.5 mm at the beginning of the next deflection (mixed model with individual cricket included as random effect: order, *P*=0.43, *F*_1,5_=0.65; direction, *P*<0.0001, *F*_1,5_=30.14, *N=*156).

The apparent length of the antennae was the same in digitized images of the length before and during bending, corroborating that bending occurred within the plane of interest (ventral–dorsal) and did not bend out of the plane (lateral–medial). The average change in total length was 1% shorter when bent, which is within the digitizing error and was not significantly different from zero (*N*=40 comparisons of traced lengths, *t*-test, *P*=0.11). This is on the order of the shortening that occurs in flagella of this species when bent; Loudon et al. (2014) have documented a decrease in length of 13% on the concave side of a sharp bend in the flagellum, but this is a localized decrease.

### Return velocity

We determined the exponential decay time constant τ*_e_* (the time to return to 1/*e* or 36.8% of the maximum deflection) to evaluate how quickly antennae returned from deflection, with a faster return having a smaller τ*_e_*. The average for all deflections of the antennae was τ*_e_*=41±13 ms (*N*=156, mean±s.d.). The time constant was significantly affected by the direction of the perturbation and the location along the antenna, but not their interaction (mixed model, direction: *F*_1,133_=8.33, *P=*0.0046; *F*_1,133_=53.35, distance: *P<*0.0001, *F*_1,133_=1.30, distance×direction: *P*=0.3, *N=*156 deflections). When deflected upwards the return was slower (τ*_e_*=45±14 ms, *N*=78) than when deflected downwards (τ*_e_*=38±12 ms, *N*=78) (mixed model, *F*_1,135_=14.55, direction: *P*=0.0002, *N*=156). When deflected closer to the base of the flagellum, the average τ*_e_* was 27 ms (5 mm, *N*=40) and τ*_e_* was 46, 49 and 44 ms for deflections farther from the base (10, 15, 20 mm, *N*=40, 40, 36, respectively). The time it took for the tip of the antenna to reverse direction as part of an oscillation (when this occurred) was significantly affected by the length of the antenna (mixed model, *F*_1,20_=7.89, *P*=0.0108, *N*=36), with longer antennae having longer periods, although this relationship is not strong (linear regression, *R*^2^=0.19, *N*=36). Additionally, length had no significant effect on τ*_e_* (mixed model, *F*_1,136_=4.40, *P*=0.73, *N*=156).

The maximum speed that the antennal tip reached during return was not dependent on sex of the cricket or direction of the deflection (mixed model, sex: *F*_1,109_=0.24, *P*=0.63, *N*=156; mixed model, direction: *F*_1,109_=0.39, *P*=0.54, *N*=156). However, the maximum speed of the antennal tip was greater when deflections were more proximal compared with distal deflections (mixed model, distance: *F*_1,109_=23.35, *P*<0.0001, *N*=156). At the most proximal distance, the average maximum speed was 897±467 mm s^−1^ (mean±s.d., *N*=156) and at the furthest deflection the average maximum speed was 213±371 mm s^−1^ (*N*=156).

### Oscillation of antennae after contacting and moving past an obstacle

**Fig. 6.**
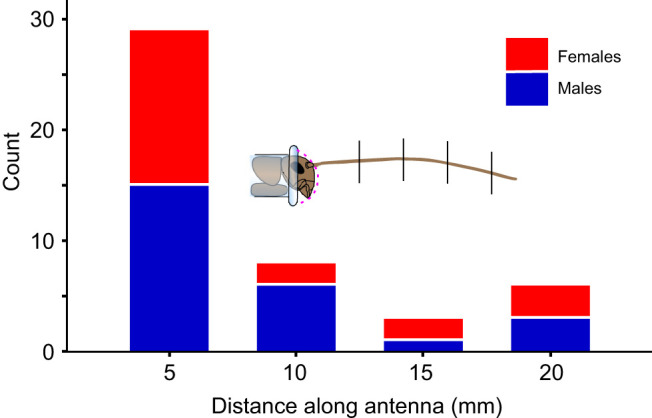
**Number of returns from deflections that oscillated (count), grouped by obstacle distance and sex.** Oscillations occur less frequently at more distal deflections (*P*<0.0001, *N*=156 videos, chi-squared) (*N*=46 videos with oscillations). Males and females are significantly different at some distances (*P*<0.0001, *N*=156 videos, Cochran–Mantel–Haenszel chi-squared). The cricket diagram illustrates distances where obstacles were located (shown on *x*-axis), measured from the proximal end of the flagellum.

To determine the extent of damping of the oscillatory behavior of the antennae following contact with the obstacle, we analyzed the number and amplitudes of oscillations. Oscillations were rare; most (71%) of the deflections (110/156) had no oscillations at all (e.g. Fig. 4A), with the antennal tips returned monotonically to their original position without any overshoot (Fig. 6). Twenty-four percent (37/156) of the deflections showed only a single reversal in direction, and the remaining 6% (9/156) showed additional oscillatory behavior (e.g. Fig. 4B). The location along the antenna where the deflection occurred significantly affected the number of oscillations of the antennal tips, with more oscillatory behavior seen when the obstacle contacted the antenna more proximally (mixed model, distance: *F*_1,152_=38.49, *P*<0.0001, *N*=156). Sex of cricket, length of antenna, all interaction terms, and the random effect of the individual cricket did not significantly affect the number of oscillations at the *P*=0.05 level.

We used chi-squared analyses to evaluate the association of oscillations (as a dichotomous variable, present or absent) with the three explanatory categorical variables (sex, distance and direction). The only explanatory variable that was associated with the presence of oscillations was distance (χ^2^_17_=14.89, *P<*0.0001, *N*=156). Results were comparable regardless of whether cricket identity was treated as a repeated measure.

**Fig. 7.**
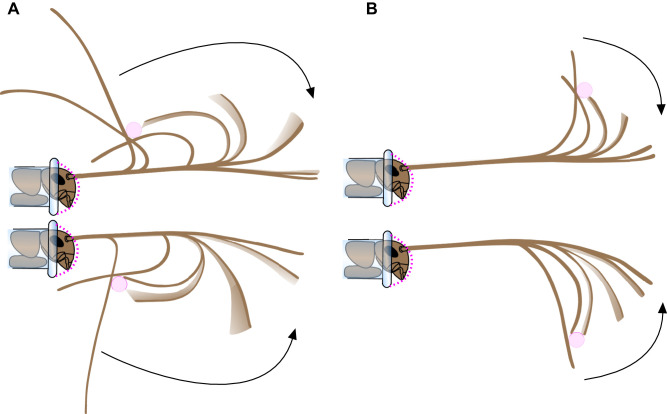
**Movement and shape changes of an antenna as it slipped past a fixed obstacle (pink) and re-straightened.** Tracings are 0.025 s apart. Dorsal (top) and ventral (bottom) deflections are shown. For a dorsal deflection, the cricket was moved down until the antenna started slipping past the obstacle; for a ventral deflection, the cricket was moved up. (A) Obstacle at 5 mm from base of antenna. (B) Obstacle at 20 mm from the base of the antenna. The portion of an antenna initially participating in the deflection is close to the proximal side of the obstacle contact. The more proximal portion acts stiff and retains its original shape. As the antenna returns, it does so progressively with the proximal portions coming to a rest before the distal part, producing an unfurling behavior, until the antenna is no longer in contact with the obstacle, at which point the distal part straightens.

Oscillations were more likely to occur when the obstacle contacted more proximal locations on the antenna, as more of the antenna was bent as it slipped past the obstacle (Fig. 7). At the most proximal deflection distance (5 mm), the tips commonly overshot the equilibrium point and then returned without further oscillations, resulting in an average of 0.5 oscillation cycles per deflection (*N*=20). At the distances of 10, 15 and 20 mm, the average number of oscillation cycles per deflection was 0.10 (*N*=20), 0.05 (*N*=20) and 0.03 (*N*=18), respectively. About twice as many oscillations of the tips of the antennae were observed when deflected up compared with deflected downwards (0.45 versus 0.21 oscillation cycles per deflection, *N*=156).

We calculated an average value for ζ of 0.6 (range 0.4–0.8; *N*=18 crickets, averaged for each cricket using 46 deflections) using the logarithmic decrement for the first half cycle using Eqn 3 with *n*=0.5 (the cycle started at a peak, and 46 videos had at least one reversal in direction; Fig. 4). We calculated a similar ζ of 0.5 (range 0.4–0.7, *N*=6 crickets, averaged for each cricket), for the subset of nine videos that had at least one complete cycle. ζ could not be calculated for the 70% of deflections that completely lacked oscillations (therefore, by definition, ζ≥1 for 70% of the antennal deflections). ζ=1.0 is considered critically damped. Thus, the cricket antennae, responding to a perturbation, behave remarkably close to critical damping.

We were able to estimate the damped natural frequency for the 46 cases of oscillation using the time between the release from the obstacle and the first reversal of direction (an estimate of half of the period). This damped natural frequency estimate was not significantly affected by sex or direction of deflection but was significantly affected by the distance of the obstacle from the base (ANOVA for distance, *F*_1,44_=10.06, *P*=0.0028, *N*=46) although the magnitude differences were small. The overall average damped natural frequency was 11±6.9 Hz (mean±s.d., *N*=46); categorizing by obstacle distance, the damped natural frequency was 14±7.4 Hz for 5 mm (*N*=29), 8±11 Hz for 10 mm (*N*=8), 6±0.3 Hz for 15 mm (*N*=3) and 7±3.8 Hz for 20 mm (*N*=6). When there was more than one cycle of oscillation, the frequency tended to increase in successive cycles. Only nine of the deflections had two or more reversals of direction that allowed for successive estimates of frequency, and eight of these oscillations had an increase in frequency with successive cycles.

### Antennal response to mechanical vibration

**Fig. 8.**
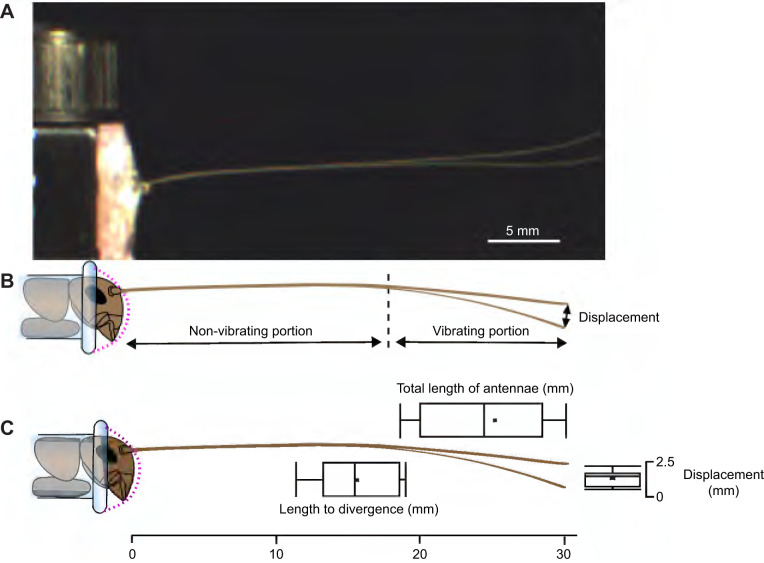
**Antennal response to mechanical vibration.** (A) Range of movement of an antenna during resonance, shown by two superimposed images; white bar shows scale. The entire length of the antennae does not participate in the vibration. (B) Diagram of non-vibrating and vibrating portions of an antenna, and displacement at the tip of the antenna. (C) Descriptive statistics of the vibrating portion of the antennae and the measured displacement at the tip. Means are indicated by ‘x’, the interquartile range is shown by the boxes, and whiskers show the range of the data. The central line in each box depicts the median (*N*=9 antennae).

When antennae were subjected to sinusoidal movements (generated by a shaker table), only about the distal third of the antenna vibrated perceptibly, with the proximal part of the antenna exhibiting no bending (Fig. 8). The average percentage of the non-vibrating proximal part of the antenna was 64% of the total length (*N*=9), and was not significantly affected by total length although there was a slight trend (regression, *P*=0.06). The total length of antennae, length of the vibrating portion of antennae and the amplitude of displacement were not significantly different between the male and female crickets (ANOVA, *F*_1,9_=0.92, *P*>0.05 for each comparison; *N*=4 females, 5 males), and therefore we combined all individuals for other analyses. Total length of the antennae and the length of the vibrating portion did not significantly affect the amplitude of the oscillations at the tip (ANOVA, *F*_1,9_=0.15, *P*>0.05, *N*=9). Oscillations only occurred at approximately 30 Hz, with an average amplitude at the tip of the antenna of 1.4±0.65 mm (mean±s.d., *N*=9), which averages 5.8% of the total length (when the amplitude is normalized to the length of the corresponding antenna, Fig. S2). The antennae did not show any perceptible movement at all (including the tips) at all other frequencies used (10, 20, 40, 50, 60, 70, 80, 90 and 100 Hz), with a temporal resolution of 500 frames s^−1^.

### Simulated deflections

**Fig. 9.**
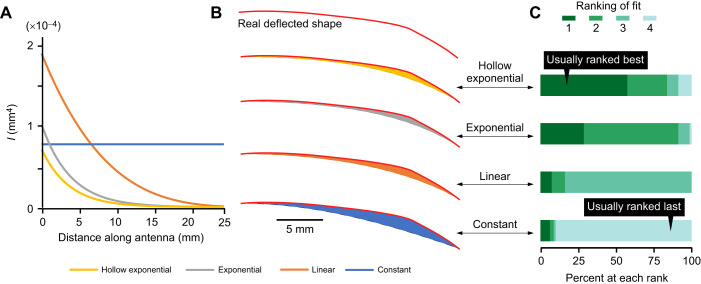
**Comparison between real and simulated bent shapes assuming different *EI* gradients.** (A) Longitudinal gradients of the second moment of area (*I*) used to estimate flexural rigidity (*EI*) gradients to predict the hypothetical bending shapes of antennae. (B) An example of a single traced deflected antenna (red line at top edges) with the four simulated deflected shapes (lower edges) corresponding to the four different *EI* gradients. The area between the two curves (colored) is used to estimate how close the simulated shapes are to the actual shape. Scale bar is 5 mm. (C) For each set of simulations (*N*=80 sets), the four different *EI* gradients were ranked in how closely they approximated the real deflected shape.

An antenna in contact with an obstacle will bend; this bent shape will be determined by the longitudinal gradient in flexural rigidity. Therefore, the (nominal) longitudinal gradient can be estimated by comparing the real shapes of antennae bent in response to obstacle contact with hypothetical simulated shapes. We compared the real shapes of antennae assuming different longitudinal *EI* gradients using the actual obstacle location and actual resting antennal shape (the latter determined in the absence of contact with an obstacle). All the models were able to produce a simulated deflection that contacted the obstacle at the defined coordinates for the individual real deflections. The fit of each model was estimated by the area between the real and simulated shapes (e.g. Fig. 9), with a smaller area indicating a better fit. The fit was dependent on the model *EI* gradient and the contact distance of the obstacle from the base of the flagellum (mixed model using area normalized to the hollow exponential model; model *EI* gradient: *F_2, 236_*=69.01, *P*<0.0001, distance: *F*_1, 236_=15.97, *P*<0.0001, *N*=360, similar results with or without cricket as a random effect).

The exponential tapering hollow model was closest to the real deflected shape for all contact point distances (Fig. S3). We found no significant difference between males and females, and no significant difference based on the direction of the deflection in the area between the simulated and the real deflected shapes (mixed model, sex: *F*_1,318_=1.78, *P=*0.18; direction: *F*_1,229_=1.05, *P*=0.31).

As another way of visualizing the data, for each real deflection, we compared the summed deviation (area) for all simulated deflections to rank the fit of the model (Fig. 9) from best (1) to worst (4). The uniform *EI* model ranked last for 90% of the deflections (90% of *N*=80 real deflections), and the exponentially tapering, hollow model ranked first most often (57% of *N=*80).

## DISCUSSION

### Minimal oscillatory behavior in antennae recovering from single perturbations

Our results show that the cricket antennae are heavily damped, as they show minimal oscillatory behavior after deflecting in response to contact with an obstacle (Movie 1). We are interested in the similarity of the antennal behavior to that expected near the critical damping regime because this could be optimal for a responsive mechanosensory structure. It is worth noting that there are sometimes solutions other than critical damping that provide the minimum time to reach equilibrium (within experimental resolution) in the slightly overdamped or slightly underdamped regimes (Lelas et al., 2023). In practice, it can be difficult to determine how close the damping of the structure is to the level of critical damping, as lack of oscillations could be a result of either critical damping or overdamping. Evidence that the magnitude of the damping is close to critical damping could be shown by a combination of an overall lack of oscillations with the ability to generate weak oscillatory behavior with particularly strong perturbations, which is what we observed. Only ∼30% of the deflections resulted in any perceptible oscillations, with a greater likelihood of oscillations occurring with more extreme deflections (obstacle contacted antenna at a more proximal location), which is consistent with being close to critically damped. Critical damping requires a balance between stored energy and energy dissipation during the return to the resting position; our results suggest that cricket antennae are close to achieving this balance, independent of the perturbation's location along the tapered antenna.

Despite the high speed of the tip movement (and therefore momentum), the number of oscillations that occurred was very few (average 0.5 cycle, *N*=156 deflections), showing that the energy is effectively dissipated (the return is damped). Dürr et al. (2022) estimate that contact sampling frequency could occur at 5 and 10 Hz for stick insects and cockroaches, which have exponential decay time constants of 200 and 100 ms, respectively. Our measured time constant for the cricket antennae was similar at 100 ms, which would allow for a rapid sampling frequency of 10 Hz. The heavy damping of the antennae ensures that these fast movements do not result in excessive vibrations. The amount of damping can be quantified by ζ (Eqn 3) using successive oscillation amplitudes; zero damping corresponds to a ζ of zero. ζ could not be calculated for the 70% of deflections that completely lacked oscillations (therefore, by definition, ζ≥1 for 70% of the antennal deflections). Our calculated average ζ of 0.6 (for the 30% of deflections with any oscillation) only includes the more extreme deflections that occurred primarily when the obstacle was close to the head (a larger proportion of the antenna was bent; Fig. 7). To put this magnitude in perspective, note that in mechanical systems ζ is usually <0.5, even for highly damped applications such as automobile shock absorber systems (Steidel, 1979, p. 206). Direction of the deflection significantly affected the number of oscillations. When bent upwards, the antennae typically had a smaller radius of curvature (Fig. 7) and a slightly faster time constant (smaller τ*_e_*), and had about twice as many oscillations, suggesting that slightly more elastic energy could be stored when the antennae is bent in that direction and the antenna has a slightly lower flexural rigidity in that direction.

**Fig. 10.**
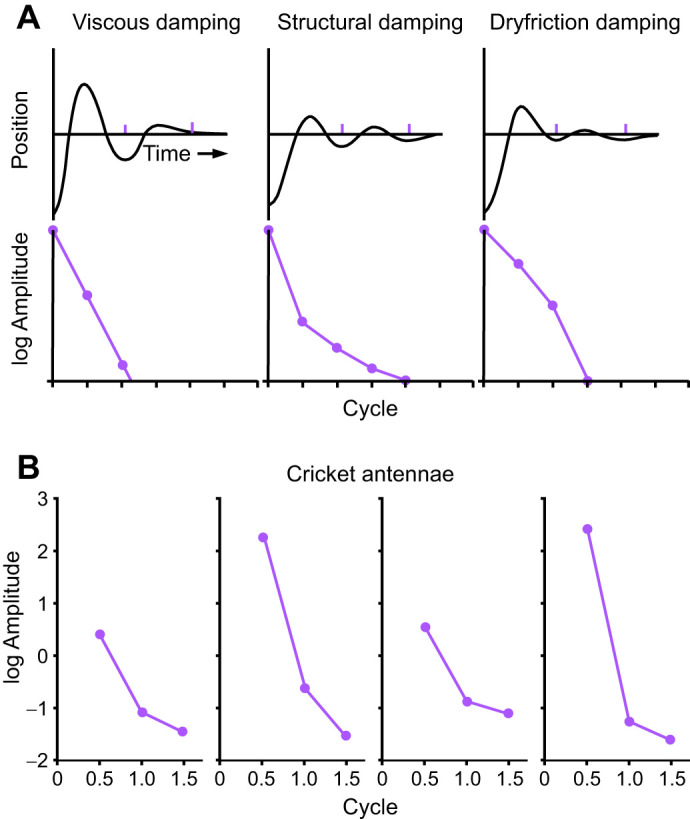
**Log-linear plots of successive amplitudes during oscillation.** (A) Log-linear plots show different patterns in the decrease in amplitude per cycle that are characteristic of the different categories of damping, resulting in a linear, concave up or concave down relationship for viscous, structural and dry friction damping, respectively. Graphs are based on Steidel (1979, fig. 6.12). (B) Four representative examples of successive amplitudes for different cricket antennae oscillating following a deflection are plotted against the number of cycles (oscillations). The amplitude ratio decreases with cycle, creating a concave up shape, which is consistent with ‘internal’ or ‘structural’, rather than viscous damping (Steidel, 1979).

The type of damping often considered first is viscous damping, in which the ratio of amplitudes in successive oscillatory cycles is constant, and the damping force is proportional to the velocity of the structure. With at least 1.5 cycles, the change in amplitude of successive (half) oscillatory cycles can be used to determine the type of damping (Steidel, 1979, fig. 6.12) (Fig. 10A). For all nine antennal deflections that had at least 1.5 cycles, our observed amplitude ratio (*P*_0_/*P*_1_) decreased with successive oscillatory cycles, which is damping behavior that is most consistent with internal (structural) damping rather than viscous damping (Steidel, 1979) (Fig. 10B). In internal damping, energy dissipation per cycle is usually independent of frequency and is dissipated at a rate greater than the square of amplitude of the oscillation (Steidel, 1979; Tongue, 2002).

### Implications for active movement of antennae

For these measurements of the passive mechanical behavior of the flagellum, we prohibited any active movement by constraining the scape and pedicel joints. Normally, the muscular attachments of the scape and pedicel (the two most basal segments on an insect antenna) allow for active movement of cricket antennae. Crickets can control their head–scape and scape–pedicel joints in a combination of dorsal–ventral or medial–lateral movements, respectively (Honegger, 1981; Krause and Dürr, 2004). This active control allows the insect to move the flagellum quickly and ‘saccade-like’ while tracking objects within their view (Honegger, 1981). Fast, active movements such as these are also influenced by damping to maintain precision of the movement and prevent overshooting a target. Although there could be functional circumstances in which oscillations need to be completely prevented (overdamping) or a rapid response is more important than potential oscillations (underdamping), these have yet to be described for insect antennae. For designs of antennae-like tactile sensors such as described in Hoinville et al. (2014), that are able to localize objects using vibrations, excess vibrations can be an obstacle for robots sensing on uneven terrain which damping might improve.

For simplification of analysis, we perturbed the antennae only ventrally or dorsally and performed a 2D analysis from the side. We did not find a need for a 3D analysis as there was not a significant difference in the apparent length of antennae while bending (during deflection), which would indicate bending perpendicular to the plane of the field of view. Dorsal–ventral movements are seen in cricket antennae during behaviors such as walking (Horseman et al., 1997) and aggressive encounters (Alexander, 1961), and are also necessary to position antennae vertically even during lateral movements. Although we restrained the muscle-driven movement of the flagellum, the crickets were alive, suggesting antennal circulation could still occur. Donley et al. (2022) demonstrated that hemolymph pressure affects the active movement of the antennae caused by a change in the pressure, such as straightening of the antennae.

### Estimation of longitudinal gradient in flexural rigidity

We evaluated the bent quasi-static shapes of the cricket antennae in contact with an obstacle to estimate the nominal longitudinal gradient in flexural rigidity. This approach has been used, for example, to identify the flexural rigidity gradient of tapered fishing rods loaded near their tips from their bent shapes (Silverman and Farrah, 2018).

Similar to Mongeau et al. (2014), who determined that the longitudinal gradient of flexural rigidity (*EI*) of antennae in cockroaches was consistent with the exponentially tapered geometry of the antennae, we found that using the taper of the cricket antennae to model a flexural rigidity gradient produced simulated curves that closely matched the real shapes of the bent quasi-static antennae. The curvature of the real bent antennae was best modeled by a beam model with an exponential taper. The ‘exponential tapering hollow cylinder’ model was usually a better predictor for the shape of the real antennae than the ‘exponential tapering cylinder’ model, although the thinning of the antennal wall had a much smaller effect on *I* than the tapering of the outer radius. Note that *I* (and therefore *EI*) is a function of the fourth power of the outer radius (Eqns 7 and 8), and therefore even a modest taper is predicted to have an extremely amplified effect on the decrease in flexural rigidity along the length of the flagellum. For example, comparing *I* at a location 10 mm from the proximal base of the flagellum, the linear model predicts a drop to 25% of the initial value of *I* (at the proximal base), whereas the exponential tapered models (both hollow and solid) predict a steep drop to 8% of the initial value of *I* (and the constant model has no decrease in *I* along the length). Moving distally along the antenna to a location 20 mm from the proximal base of the flagellum, the linear taper model now predicts a steep drop to 3% of the proximal value of *I*, whereas the exponential taper models predict even lower values of approximately 0.7% of the proximal value of *I*.

Several species of arthropods exhibit both tapering and a flexural rigidity gradient along the proximal–distal axis of their antennae. For example, Dirks and Dürr (2011) found a nonlinear taper in the stick insect, Mongeau et al. (2014) described an exponential taper in *Periplaneta*, and Sandeman (1989) reported a linear taper in crayfish. In all of these cases, the flexural rigidity measurements decreased nonlinearly along the length (which would be expected for a linear or exponential taper). Because bending in a flagellum takes place at the joints between the flagellomeres (Loudon et al., 2014; Sandeman, 1989), these nominal estimates of flexural rigidity presumably reflect the structural properties exhibited by the complex folding of the arthrodial membranes, the sizes of which are determined by the taper. The proximal–dorsal pattern in flagellomere length shows tremendous variation (Loudon et al., 2014; Mongeau et al., 2014; Sandeman, 1989). These differences in flagellomere length distribution do not appear to disrupt the consistent biomechanical trend across taxa of a nonlinear decrease in flexural rigidity along the proximal–distal axis for filiform antennae.

### Functional consequences of a steep flexural rigidity gradient to a sensory structure

Flexural rigidity is the resistance of a structure (e.g. a beam) to bending, which determines the amount of force needed to be applied to a point on the beam, to displace the beam a given distance. Greater flexural rigidity requires more force to achieve a given degree of deflection. Tapering allows the distal parts of the beam to be more flexible (smaller *I*) than the more proximal parts of the beam, resulting in localized greater curvature near the contact point rather than at the support, as with a uniform beam. For many insects, bending of the antennae elicits a behavioral response, such as reaching for an obstacle to climb, initiating a rapid escape, or changing the heading angle to remain in contact with a wall (Camhi and Johnson, 1999; Comer et al., 2003; Schütz and Dürr, 2011; for review, see Dürr et al., 2022).

The flexural rigidity gradient along an antenna will affect how the forces and moments are mechanically transmitted to the sensory organs close to the base of the antenna when the antenna deflects at different distances, potentially allowing for information about distance from obstacles to be derived, similar to localization shown in rat whiskers (Quist and Hartmann, 2012). Currently, it is unclear whether insects use the slip of their antennae along objects to gather positional information based on changes in antennae contact curvature (for review, see Dürr et al., 2022). Friction between an antenna and an obstacle is likely to be anisotropic (direction-dependent) because the sensory hairs are often angled and projecting in a distal direction (including on the flagellum of *A. domesticus* crickets; Loudon et al., 2014), and therefore such an antenna will slide more easily along a contact point in a proximal to distal direction. This directional angling of hairs on a tactile antenna will facilitate the smooth and rapid movement of an antenna deflecting around an obstacle such as described in the present study for cricket antennae. This change in moment might also help crickets determine distance information as their antennae move along obstacles. Similarly, Mongeau et al. (2013) demonstrated by sensory hair removal that when the distal end of a cockroach antenna is dragged along a surface during wall-following, its orientation will be influenced by the presence of the angled sensory hairs. Using physical models of cockroach antennae that differed in their flexural rigidity gradients, Mongeau et al. (2014) showed a better correlation between bend location and wall distance with models that had steeper flexural rigidity gradients, suggesting that tapering would increase preview distance in cockroaches, potentially enhancing their escape reaction time. For whiskered animals such as rats, localized whisker bending aids in object distance estimation (Hires et al., 2013; Williams and Kramer, 2010; for review, see Dürr et al., 2022). In house crickets, *EI* measurements at the proximal portion of the antennae showed no difference between dorsal and ventral directions (Kellogg, 2007), though we observed a slight difference in bending curvature in the dorsal and ventral directions. It is unclear whether these small dorsal–ventral differences in flexural rigidity would influence object distance detection in these different directions.

Tapered antennae have less mass distally, and therefore less momentum that will diminish the likelihood of oscillations. Thus, tapering is an example of a geometric trait that can lead directly to increased damping. An application of this is the use of tapered steel beams as simple devices to damp earthquake forces in bridges and buildings (Tyler, 1978).

As the antennae reconfigure into their original shape, they do so in a progressive manner. The proximal parts of the antenna stop moving before the distal parts, creating an ‘unfurling’ behavior of the antennae (Fig. 7) as the more distal parts of the antennae straighten and stop vibrating. Unfurling is a novel mechanical behavior that has not previously been described for mechanosensors. Initially, while the antenna is still in contact with and slipping past the obstacle, there is a localized area of steep curvature which is proximal to the point of contact, and travels distally as slip occurs. Once released from the obstacle, the localized bend straightens out, like a hinge (Fig. 7). There are few analogs to this behavior apart from unfurling and refurling of flexible structures with localized areas of high curvature, such as the use of tape springs (Seffen and Pellegrino, 1999) or ridge springs (Seffen, 2020), which have applications in space engineering (e.g. Baudasse et al., 2017). The desired behavior of a straightening tape spring from a localized bend is to reach its final state in a minimal time without overshoot or oscillation (Lyle and Horta, 2012). In contrast with a cricket antenna, a tape spring is typically uniform along its length and therefore has a simpler geometry and a constant flexural rigidity.

Another consequence of the steep flexural rigidity gradient observed in antennae is that it protects the antennae from breaking, as the flexible tips deflect off or around obstacles. Damaging the antennae is important to avoid as it can lead to a decreased tactile reach, and can incur fitness costs with regards to mating, as males with shorter, broken antennae are less likely to reproduce (Khadka et al., 2012).

### Frequency response of tapered antennae

We observed that the frequency of successive oscillations in an individual antenna increased slightly. This is consistent with a decreasing length of the antenna participating in the oscillation over time. Also note that a higher frequency (for a given ζ) will lead to a faster decrease in oscillation amplitude with time (cycles are shorter). Similarly, when driven at the resonant frequency, the entire length of the antennae did not deflect, with the proximal part behaving like a rigid body (Fig. 8). This is in contrast with the oscillatory behavior that would be expected of a beam with uniform flexural rigidity. When driven at frequencies other than resonance, neither the tip nor the base of the flagellum moved appreciably. Although in the case of cricket antennae the damping is related to the tapering of the antennae, in other applications, different approaches or structures can be built to act like a mechanical filter, such as a washing machine drum with a tuned mass-damper attached that prevents vibration of the machine's drum (Argentini et al., 2013). Currently, there are no examples of damping in insect antennae that could be described as tuned mass-dampers.

### Contributing factors to damping in insect antennae

For a mechanosensory structure such as a cricket antenna, being close to critical damping could be functionally significant because it allows the structure to return to a resting condition as rapidly as possible, to be ready for new tactile sensory input. Damping can also ‘tune’ a sensory response to a specific stimulus, such as mosquito (*Aedes aegypti*) antennae behaving as simple forced damped oscillators when driven by sound vibrations, leading to greater responses by male mosquitoes to the frequency of female flight sounds (Göpfert et al., 1999). Damping of the antennae is strongly associated with the endocuticle in finite element modeling of antennae, with tapering having a smaller effect than the amount of endocuticle (Rajabi et al., 2018). Water content in the endocuticle, which is difficult to measure (Klocke and Schmitz, 2011), is hypothesized to lead to greater damping (Rajabi et al., 2018 and reviewed in Dürr et al., 2022). In further support of this interpretation, desiccation of the antennae of stick insects resulted in a shift from overdamping to underdamping (Dirks and Dürr, 2011). The role of endocuticle in damping in cricket antennae is unclear.

### Conclusion

The functional design of a tapered mechanosensor is of interest as the basic design concepts can have applications in the development of technological sensors or microelectromechanical systems. Antennae return rapidly to their original shape without oscillation owing to being near critical damping and exhibit unfurling behavior. Damping can help reduce excess noise transmitted to the mechanosensory organs, and localized bending is an important feature of sensor function that follows directly from the tapered morphology.

## Supplementary Material

10.1242/jexbio.249243_sup1 Supplementary information
