# Supplementary material for: Tapered insect (Acheta domesticus) antennae have rapid damped return with minimal oscillation after perturbation
Source: J Exp Biol. 2025 May 6;228(9):jeb249243. doi: 10.1242/jeb.249243 (PMC12079660; doi:10.1242/jeb.249243)
Supplement: Supplementary information [file jexbio-228-249243-s1.pdf]

Supplementary Materials and Methods

Normalizing antennal geometry

Crickets vary in the length of their antennae, but in practice it is not possible to know with certainty if a shorter antenna is just naturally shorter (such as fewer flagellomeres or shorter flagellomeres) or if the distal part of the flagellum was broken as a result of interactions with another cricket or became abraded in the environment, or both (naturally shorter and some abrasion). Crickets do not have a fixed number of flagellomeres. Normalizing the geometry of each antenna (width/length relationship) by its (1) width at the base of the flagellum and (2) total length, can lead to an artificial spread in the values at the distal end (Figure S1).

Additional details in the use of Elastica2D

Elastica2D is quasi-static (movement is slow enough to approximate equilibrium). It uses an iterative numerical approach to simulate bending a structure until it meets the obstacle coordinates as described in the methods. The "nodes" are rigid links connected by torsional springs (Quist and Hartmann, 2012) which is reminiscent of the behavior of cricket flagella which bend at the joints between flagellomeres (Loudon et al., 2014). We followed methods similar to (Quist and Hartmann, 2012) who rotated a whisker against a stiff peg to obtain bent shapes during deflection, then compared these shapes to the predicted simulations. We used still images (single frames) during bending while the micromanipulator was moving the cricket very slowly (see methods). The coordinates of the shape of each antenna was collected by tracing it using a curvilinear line tool, overlaying a grid on the traced line to divide the trace into individual elements using Canvas X (Canvas GFX, Inc.), and exporting the coordinate points for the nodes. Since the length of the antennae varied, the number of nodes was variable (the real distance between nodes was constant between antennae). On average there were roughly 200 nodes per antenna. Elastica2D has a subroutine which ensures the points are equally spaced along the length of the beam. In addition, the program makes the following assumptions:

1. The applied force is normal to the surface of the bending structure.
2. There is no friction. Force is assumed to be applied normal to the beam.
3. The first segment is colinear with the axis and the base is centered at 0,0.
4. Nodes are equidistant.
5.The deflection occurs in 2-dimensional spac

Journal of Experimental Biology • Supplementary information

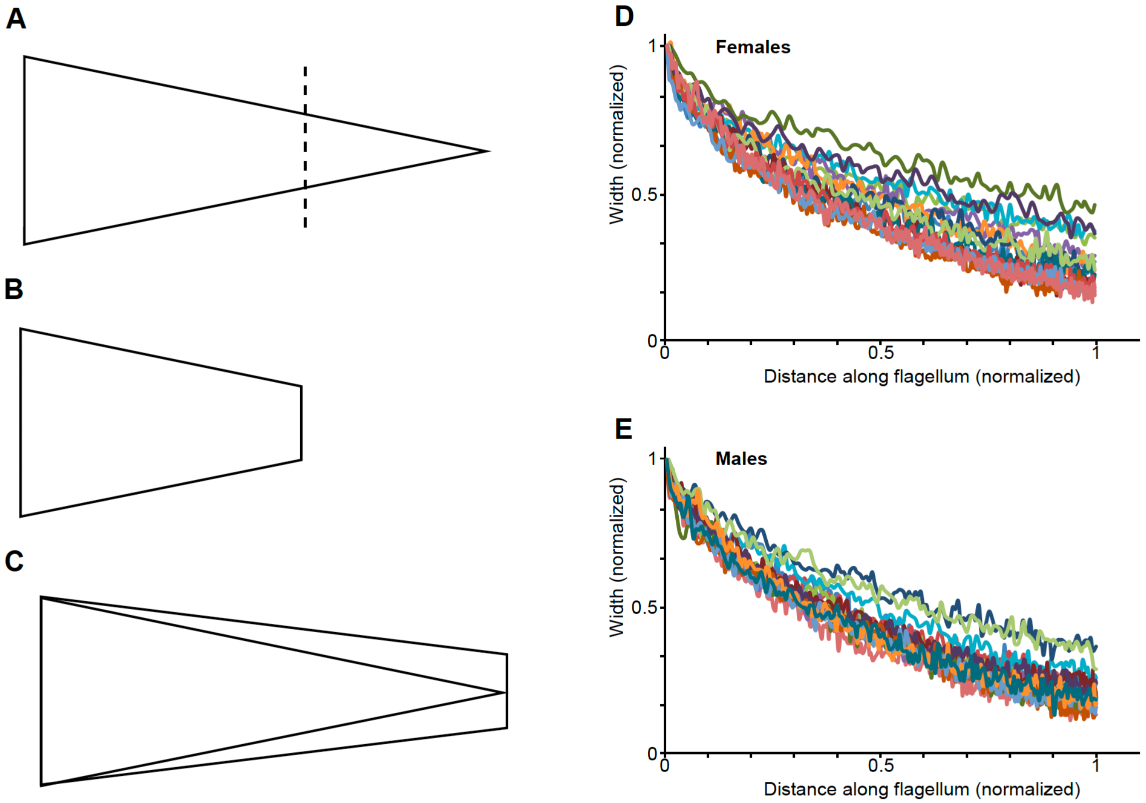

Fig. S1. Geometry of cricket antennae (length-width) following normalization. A. A diagram of a tapered antenna (linear taper shown for simplicity). B. A shorter antenna that has the same taper in real-world measurements; its length compared to A is indicated by the dashed line in A. C. Normalizing the two antennae (A and B) to the same length makes the B antenna appear to be wider at the distal end, although it is identical in width at every real distance. D. Female data from Loudon et al. (2014) and graphed in Fig. 2 recalculated by normalizing each antenna by its total length (each line is an individual antenna). E. Male data from Loudon et al. (2014) and graphed in Fig. 2 recalculated by normalizing each antenna by its total length.

Journal of Experimental Biology • Supplementary information

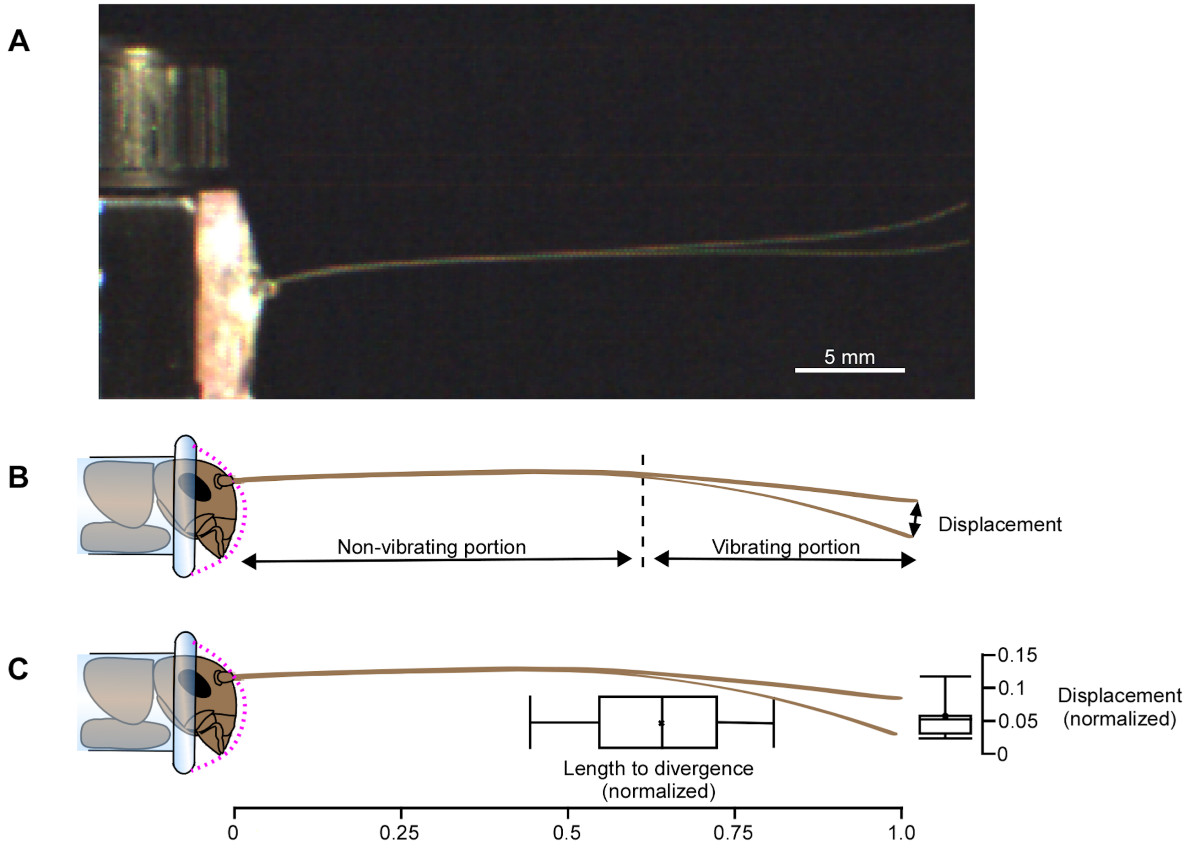

Fig. S2. Antennal response to mechanical vibration (normalized by antennal length). (A) Range of movement of antennae at maximum amplitude during resonance, shown by two superimposed images; white bar shows scale. The entire length of the antennae does not participate in the vibration. (B) Diagram of non-vibrating and vibrating portions of an antenna, and displacement amplitude at the tip of the antenna. (C) Descriptive statistics of the vibrating portion of the antennae and the measured amplitude at the tip, both normalized by antennal length. X-marks indicate means, the interquartile range is shown by the boxes, and whiskers show the range of the data. The central line in each box depicts the median (N = 9 antennae). This figure is a normalized version of Fig. 8).

Journal of Experimental Biology • Supplementary information

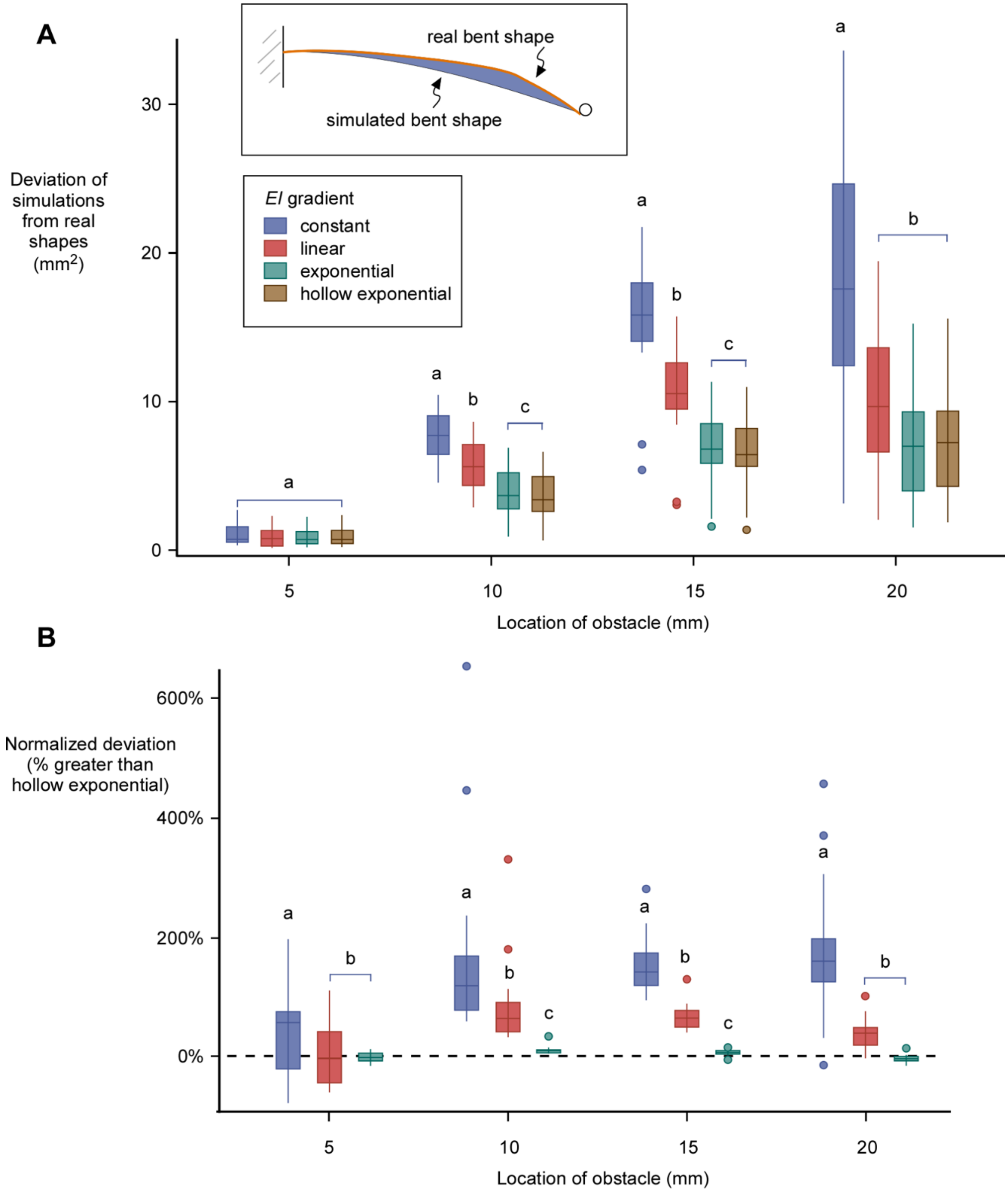

Fig. S3. Comparison between real bent shapes and simulated bent shapes assuming different *EI* gradients. The box plots show the total range of the data (vertical lines), the median is indicated by a horizontal line, and the box shows the quartiles. Circles indicate outliers; all outliers were included in the statistical analyses (see Methods section for convention used to identify outliers). (A) The deviation of each

Journal of Experimental Biology • Supplementary information

simulated antennal shape from the real deflected antennal shape was estimated by the area between them (see inset), thus the greater the area, the worse the fit. Each simulated antennal shape was generated by starting with the real undeflected antennal shape (at rest) and predicting how it would bend (deflect) assuming a particular *EI* gradient and the real location of the obstacle causing the bending (indicated by a circle in the inset) (see text for more details). For each obstacle location distance (measured from the proximal end of the flagellum), 10 different crickets were used (5 males and 5 females), with deflections in both directions (dorsal and ventral), for a total of 20 deflected real shapes for each location distance. For each location, a mixed model was used to evaluate whether there was a significant difference in the fit between different *EI* gradients (estimated by area as described above); cricket identity was included as a random factor when significant (at the $P = 0.05$ level), and when there was an overall significant difference between the four gradients, all pairwise comparisons were made using the Tukey-Kramer posthoc adjustment. The letters (a, b, c) indicate significant differences between different *EI* gradients ($P < 0.05$ after adjustment). (B) Deviations between real and simulated shapes for three of the *EI* gradients (constant, linear, and exponential) were normalized with respect to the deviation estimated using the hollow exponential *EI* gradient because the latter is the closest approximation to antennal geometry. The formula is $(\text{Area}_{EI\text{ gradient}} - \text{Area}_{EI\text{ gradient, hollow exponential}})/(\text{Area}_{EI\text{ gradient, hollow exponential}})$. Mixed models and all pairwise comparisons were performed on the normalized values for each location using the same methods and conventions described above.

Journal of Experimental Biology • Supplementary information

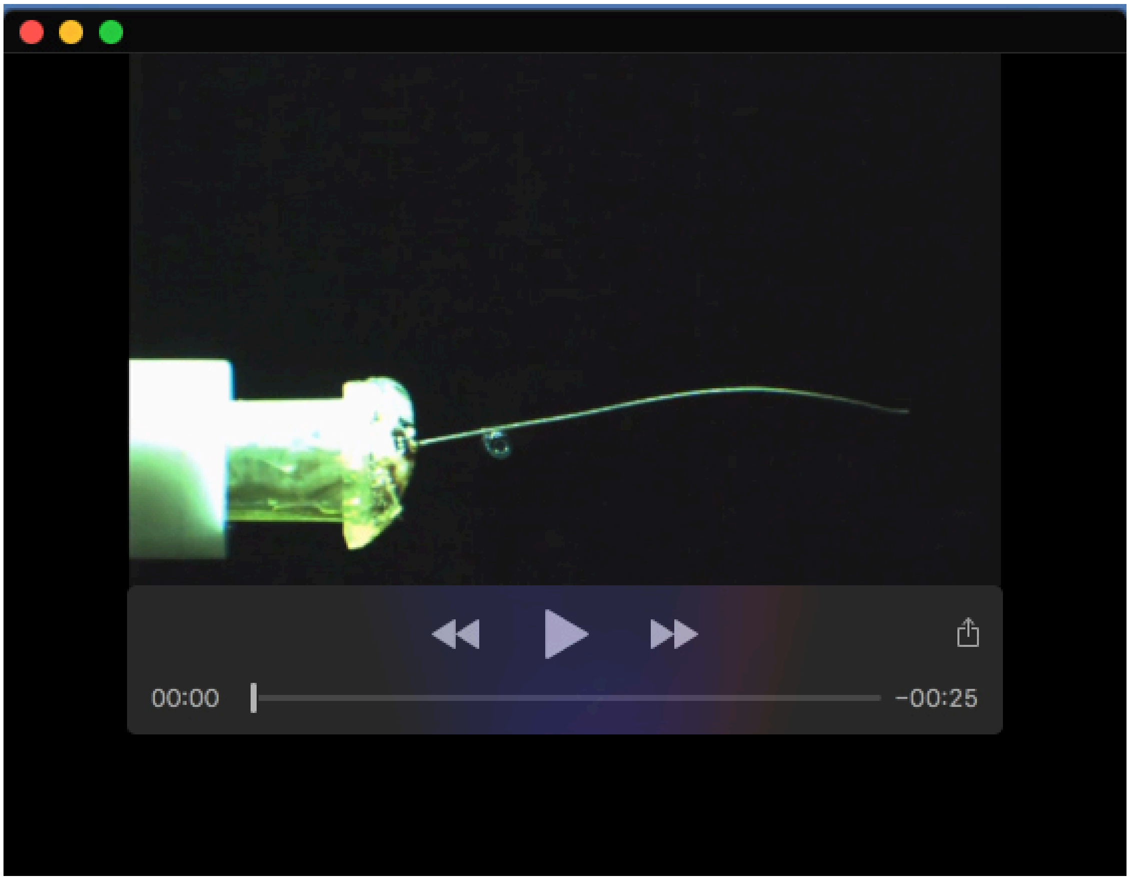

Movie 1. A single upwards deflection of an antenna of a live, restrained cricket. Obstacle is at a distance of 5 mm from the proximal end of the flagellum. Playback speed is in real time.

Loudon, C., Bustamante, J. J. and Kellogg, D. W. (2014). Cricket antennae shorten when bending (*Acheta domesticus* L.). *Frontiers in Physiology, Section Invertebrate Physiology* **5**, 1-9.

Quist, B. W. and Hartmann, M. J. Z. (2012). Mechanical signals at the base of a rat vibrissa: the effect of intrinsic vibrissa curvature and implications for tactile exploration. *Journal of Neurophysiology* **107**, 2298-2312.
